# Impact of risk factors for major cardiovascular diseases: a comparison of life-time observational and Mendelian randomisation findings

**DOI:** 10.1136/openhrt-2021-001735

**Published:** 2021-09-13

**Authors:** Lars Lind, Martin Ingelsson, Johan Sundstrom, Johan Ärnlöv

**Affiliations:** 1 Department of Medical Sciences, Uppsala University, Uppsala, Sweden; 2 Department of Public Health and Caring Sciences/Geriatrics, Uppsala University, Uppsala, Sweden; 3 School of Health and Social Studies, Dalarna University, Falun, Sweden; 4 Division of Family Medicine and Primary Care, Department of Neurobiology, Care Sciences and Society (NVS), Karolinska Institutet, Stockholm, Sweden

**Keywords:** heart failure, atrial fibrillation, epidemiology, stroke, coronary artery disease

## Abstract

**Background:**

This study compared the strength and causality of associations between major risk factors for cardiovascular disease (CVD) and the four major CVDs: myocardial infarction, ischaemic stroke, heart failure and atrial fibrillation. Both a long-term follow-up in an observational cohort and Mendelian randomisation (MR) were used for this aim.

**Methods:**

In the Uppsala Longitudinal Study of Adult Men study, 2322 men, all aged 50 years, were assessed for CVD risk factors and then followed for four decades regarding incident CVDs. The two-sample MR part used public available Genome-Wide Association Study (GWAS) data.

**Results:**

In multivariate analyses, systolic blood pressure was overall by far the most important risk factor, since it was related to all four CVDs, both in observational and MR analyses. Body mass index was the second most overall important risk factor, being linked to all four CVDs, except ischaemic stroke, both in observational and MR analyses. Smoking was an important risk factor for ischaemic stroke and heart failure, both in observational and MR analyses, while low-density lipoprotein-cholesterol mainly was related to myocardial infarction. Diabetes was mainly a causal risk factor for incident myocardial infarction and heart failure. Neither HDL-cholesterol nor triglycerides were of major importance as risk factors in these multivariable models.

**Conclusion:**

By combining long-term observational data with genetic data, we show that the impact and causal role of specific established cardiovascular risk factors varies between different major CVDs. Systolic blood pressure was causally related to all four cardiovascular outcomes and was therefore, overall, the most important risk factor.

**Key questions**

**What is already known about this subject?**

- A risk factor for different cardiovascular diseases has previously been identified.

**What does this study add?**

- This study compares observational data with Mendelian randomisation data for each traditional risk factor regarding myocardial infarction, stroke and heart failure.

**How might this impact on clinical practice?**

- It is of great importance to know which of the risk factors found in observational studies that are causal.

## Introduction

Our understanding of the strength of specific cardiovascular risk factors for different cardiovascular diseases (CVDs) is predominantly based on data from longitudinal studies with around 10 years follow-up.^1^ From these data, risk scores for coronary heart disease (CHD),^2^ stroke,^3^ heart failure^4^ and atrial fibrillation^5^ have been constructed with data from the Framingham Heart Study, as well as other studies.^6 7^ A common view from these studies is that these four major CVDs share the same risk factors, like systolic blood pressure (SBP), diabetes, high low-density lipoprotein (LDL) and low high-density lipoprotein (HDL)-cholesterol, obesity and smoking, but that the strength of the specific risk factors varies between the different CVDs.

In epidemiological settings, where randomised control trials are often difficult to implement, Mendelian randomisation (MR) analysis can be used to assess causality.^8^ The method uses randomly distributed genetic variants that are robustly associated with a risk factor (the exposures in this case) as instrumental variables to test for causal effects on an outcome. In recent years, a number of MR studies have investigated the causal role of cardiovascular risk factors for the development of different CVDs.^9–14^ However, no study has in a systematic fashion investigated all the traditional risk factors versus the four major CVDs; CHD, ischaemic stroke, heart failure and atrial fibrillation. Furthermore, in most Genome-Wide Association Studies (GWAS) performed for CVDs, a majority of the cases were elderly. Thus, in these studies, the genetic impact on the disease would have been present for many decades prior to disease onset. Thus, if the results from an MR study of risk factors on CVDs should be compared with observational data, ideally, the observational data should have a long follow-up period.

This study aims to fill a gap in our knowledge on shared and specific causal risk factors for the four major CVDs. First, the strength of the traditional risk factors for CHD, ischaemic stroke, heart failure and atrial fibrillation were investigated in a longitudinal study with 40 years follow-up (the Uppsala Longitudinal Study of Adult Men, ULSAM study). The impact of the risk factors was compared between the different CVDs, and also compared with their possible causal role evaluated by MR analyses.

## Methods

In 1970–1974, 2322 men all aged 50 years living in the city of Uppsala, Sweden, were investigated as part of the ULSAM (https://www.pubcare.uu.se/ulsam/). Of the invited individuals, 82% accepted to participate.

### Traditional risk factors

The baseline examination of ULSAM in the early 70s when participants were 50 years old has been described in detail previously.^15^ Fasting blood samples were drawn in the morning after an overnight fast. Serum levels of cholesterol and triglycerides, and HDL were assayed by enzymatic techniques. Friedewald’s formula was used to calculate LDL-cholesterol. Moreover, fasting blood glucose was measured using an oxidase method. Supine SBP and diastolic blood pressures were measured twice in the right arm after 10 min rest, and means were calculated. Data on smoking status at baseline were based on a questionnaire.

### CVD diagnosis

Data on cause-of-death and hospitalisations were retrieved from the Swedish Cause of Death Register and the Swedish Hospital Discharge Register, respectively. The four major CVDs were defined as: acute myocardial infarction (International Classification of Diseases, Revision 8 (ICD-8) code 410, ICD- 9 code 410 or ICD-10 code I20), ischaemic stroke (ICD-8 codes 431, 433–436, ICD-9 code 431, 433–436, ICD-10 code I63-I66), heart failure (ICD-8 codes 427.00, 427.10, 428.99, ICD-9 code 428, and ICD-10 code I50, as well as hypertensive heart disease with heart failure (ICD-10 code I11.0)) and atrial fibrillation (ICD-8 code 427.9, ICD-9 code 427D and ICD-10 code I48). In addition, we also used data from the ECG recordings at the physical re-examinations at ages 60, 70, 77, 82 and 87 years to identify additional cases of incident atrial fibrillation. The accuracy of myocardial infarction, stroke and atrial fibrillation in the Swedish registers have been deemed of high quality.^16^ As the heart failure diagnosis has shown less validity, we performed additional chart review based validation of heart failure events as previously described.^17^ There was no lost to follow-up. The baseline examination was performed in 1970–1974 and data on cause-of-death and hospitalisations were obtained until 31 December 2014, giving four decades of follow-up.

### Statistics

In the observational part of the study, the distribution of triglycerides was skewed to the right, therefore, an ln-transformation was performed to promote a normal distribution. Thereafter, all continuous risk factors were Z-transformed to enable comparisons of strengths of associations between each risk factor and outcome. We used Cox proportional hazards regression models to evaluate the associations between the risk factors and the four outcomes. First, each risk factor–outcome pair was evaluated in a separate model (univariate). Thereafter, all seven risk factors entered the same model (multivariate). Age at baseline and sex were the same in all subjects. The proportional hazard assumption was met for all reported significant results when evaluated by visual inspection of Kaplan-Meier curves.

In the MR part of the study, summary data from published GWAS studies regarding each exposure–outcome pair were used in a two-sample approach: For the lipids, data from the Global Lipids Consortium were used.^18^ Data from the GIANT consortium were used for body mass index (BMI).^19^ Data from the DIAGRAM (DIAbetes Genetics Replication And Meta-analysis) consortium were used for diabetes.^20^ For current smoking and SBP, available GWAS from UK biobank (http://www.nealelab.is/uk-biobank) and own calculations using UK biobank were used,^21^ respectively. Regarding the outcomes, data from the CARDIoGRAMplusC4D Consortium were used for CHD,^22^ data from the METASTROKE consortium were used for ischaemic stroke,^23^ GWAS from the HERMES consortium was used for heart failure^24^ and for atrial fibrillation the recent large GWAS study by Roselli *et al* was used.^25^

As instrument for the exposure, only independent genetic variants (single nucleotide polymorpisms (SNPs)) with p<5×10^−8^ were used. Independency of these variants was evaluated by the clump command in the package MRbase in R (V.3.6.1).

Just as in the case of the observational data, the risk factors were evaluated one by one (univariate), as well as all seven together (multivariate) in the MR models.

The inverse-variance weighted (IVW) test was considered as the primary MR test, and MR Egger and the weighted median test as sensitivity tests. Both IVW and MR Egger were used for both the univariate and multivariate models, while the weighted median test was only used in the univariate setting.

In order to define a significant association as causal, we also demanded the causal estimates of the MR Egger test and the weighted median test to be in the same order as when using IVW, and the weighted median test to show p<0.05.

STATA V.16 (Stata) was used for calculations if not stated otherwise.

## Results

Baseline characteristics in the ULSAM study at age 50 are given in table 1.

**Table 1:** Baseline characteristics in the ULSAM study at age 50

Variable	Mean (SD) or proportion (%)
Fasting glucose (mmol/L)	5.5 (0.9)
Triglycerides (mmol/L)	1.93 (1.24)
HDL (mmol/L)	1.36 (.38)
LDL (mmol/L)	5.26 (1.19)
BMI (kg/m^2^)	25.0 (3.1)
SBP (mm Hg)	133 (18)
DBP (mm Hg)	83.7 (11.2)
Diabetes (%)	4.1
Current smoking (%)	51

BMI, body mass index; DBP, diastolic blood pressure; HDL, high-density lipoprotein; LDL, low-density lipoprotein; SBP, systolic blood pressure; ULSAM, Uppsala Longitudinal Study of Adult Men.

During the follow-up period, 552 incident cases of myocardial infarction, 349 cases of ischaemic stroke, 405 cases of heart failure and 556 incident cases of atrial fibrillation occurred.

### Myocardial infarction

**Figure 1:**
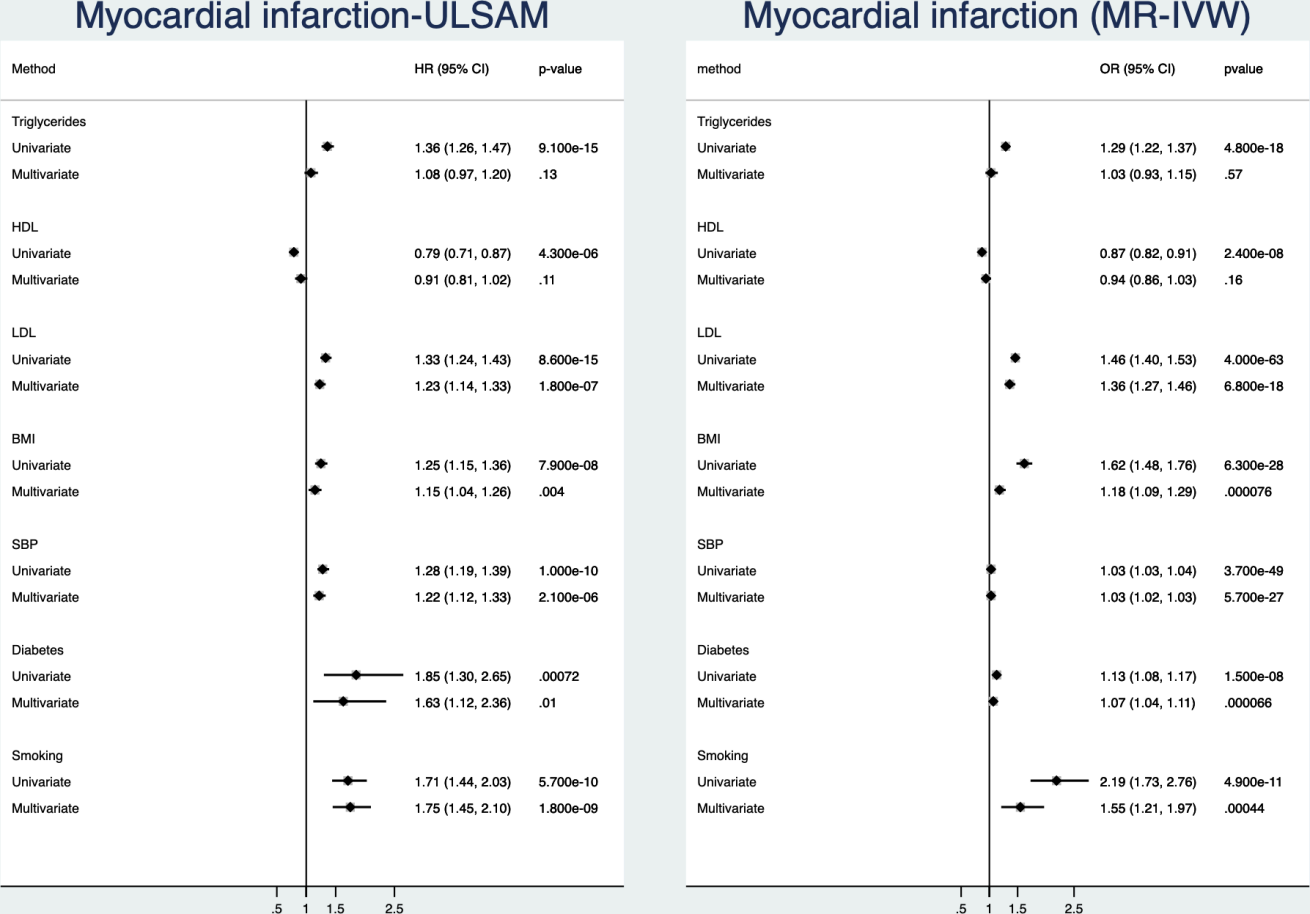
Relationships between traditional risk factors and incident myocardial infarction. Left is results from the ULSAM longitudinal study with 40 years follow-up and right is results from two-sample Mendelian randomisation (MR). Results are given both for a univariate (risk factors analysed one by one) and a multivariate model (including all risk factors in the same model). BMI, body mass index; HDL, high-density lipoprotein; IVW, inverse-variance weighted; LDL, low-density lipoprotein; SBP, systolic blood pressure; ULSAM, Uppsala Longitudinal Study of Adult Men.

(Figure 1, online supplemental table 1) In the observational part of the study, all of the seven evaluated risk factors were related to incident myocardial infarction when analysed one by one (univariate). In the multivariate model, HDL-cholesterol and triglycerides were no longer significant. LDL, SBP and smoking were the risk factors being most closely related to incident myocardial infarction, with similar low p values.

10.1136/openhrt-2021-001735.supp1 Supplementary data

In the MR part of the study, using IVW, all of the seven evaluated risk factors were related to CHD in the univariate analyses. However, the additional MR Egger and weighted median analyses gave evidence against that HDL-cholesterol is causally related to CHD. In the multivariate MR model, HDL-cholesterol and triglycerides were no longer significant. In this analysis, LDL and SBP were the two risk factors being most closely related to incident myocardial infarction.

### Ischaemic stroke

**Figure 2:**
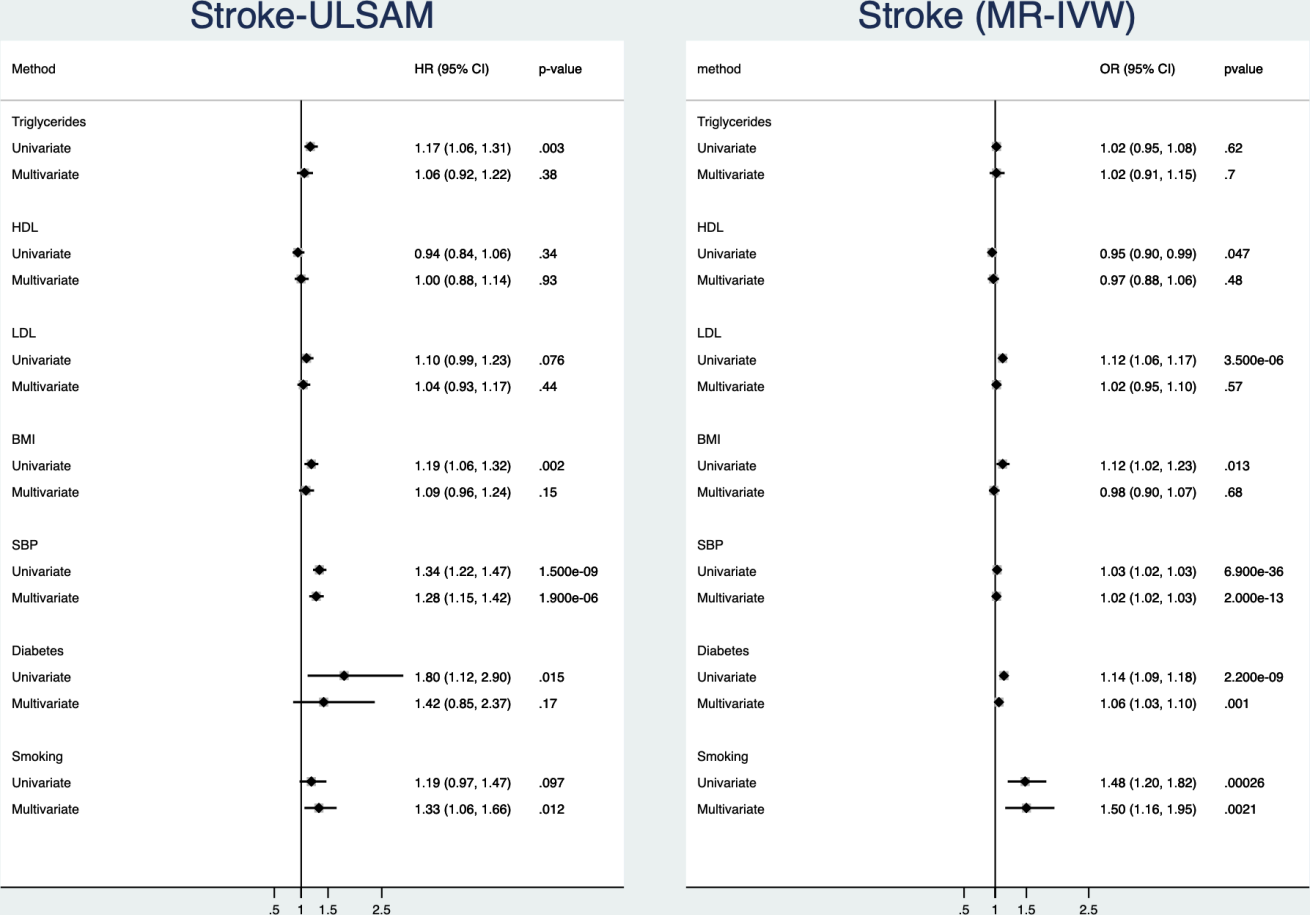
Relationships between traditional risk factors and incident ischaemic stroke. Left is results from the ULSAM longitudinal study with 40 years follow-up and right is results from two-sample Mendelian randomisation (MR). Results are given both for a univariate (risk factors analysed one by one) and a multivariate model (including all risk factors in the same model). BMI, body mass index; HDL, high-density lipoprotein; LDL, low-density lipoprotein; SBP, systolic blood pressure; ULSAM, Uppsala Longitudinal Study of Adult Men.

(Figure 2, online supplemental table 2)In the observational part of the study, triglycerides, BMI, SBP and diabetes were related to incident ischaemic stroke in the univariate analyses. In the multivariate model, only smoking and SBP were significantly related to ischaemic stroke. Of those two, SBP was by far the risk factor being most closely related to ischaemic stroke.

In the MR part of the study, using IVW, all of the evaluated risk factors were related to CHD in the univariate analyses but triglycerides. In the multivariate MR model, only smoking, diabetes and SBP were significantly related to ischaemic stroke. Of those three, SBP was by far the risk factor being most closely related to ischaemic stroke.

### Heart failure

**Figure 3:**
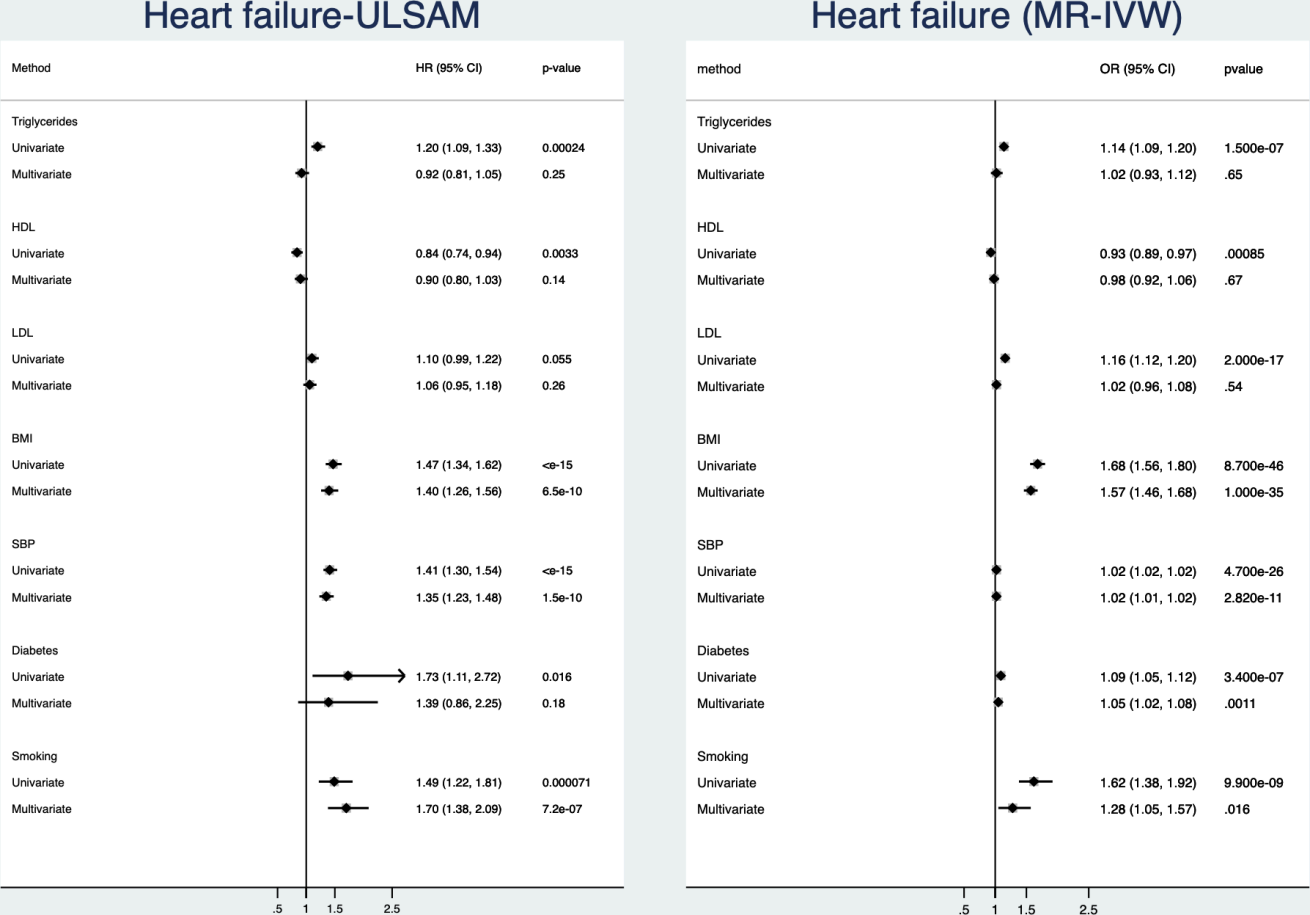
Relationships between traditional risk factors and incident heart failure. Left is results from the ULSAM longitudinal study with 40 years follow-up and right is results from two-sample Mendelian randomisation (MR). Results are given both for a univariate (risk factors analysed one by one) and a multivariate model (including all risk factors in the same model). BMI, body mass index; HDL, high-density lipoprotein; LDL, low-density lipoprotein; SBP, systolic blood pressure; ULSAM, Uppsala Longitudinal Study of Adult Men.

(Figure 3, online supplemental table 3) In the observational part of the study, all risk factors but LDL were related to incident heart failure in the univariate analyses. In the multivariate model, BMI, smoking and SBP were significantly related to heart failure. Of those three, SBP and BMI were the risk factors being most closely related to heart failure.

In the MR part of the study, using IVW, all of the evaluated risk factors were related to heart failure. In the multivariate MR model, smoking, diabetes, BMI and SBP were significantly related to heart failure. Of those four, BMI was the risk factor being most closely related to heart failure, followed by SBP.

### Atrial fibrillation

**Figure 4:**
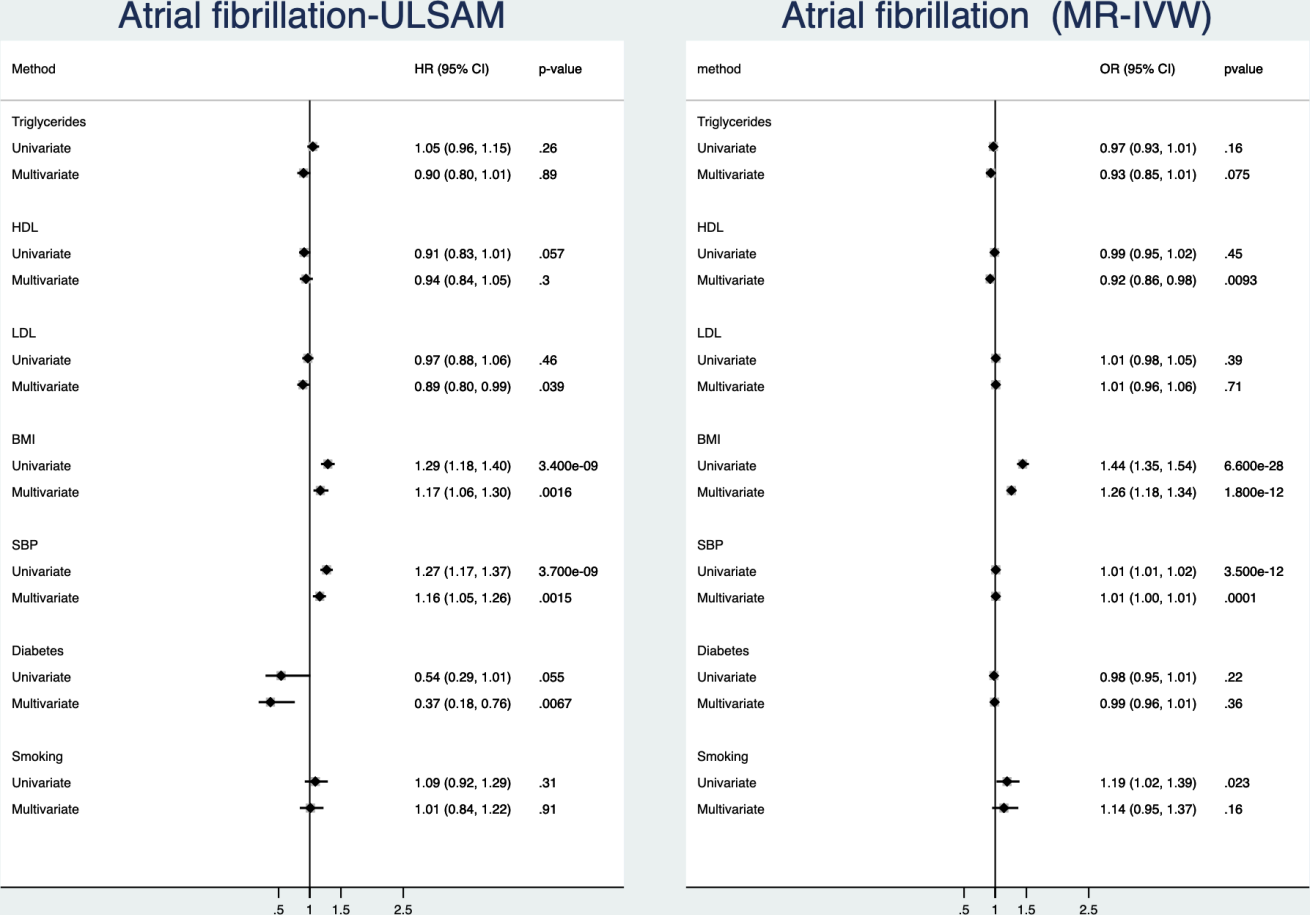
Relationships between traditional risk factors and incident atrial fibrillation. Left is results from the ULSAM longitudinal study with 40 years follow-up and right is results from two-sample Mendelian randomisation (MR). Results are given both for a univariate (risk factors analysed one by one) and a multivariate model (including all risk factors in the same model). BMI, body mass index; HDL, high-density lipoprotein; LDL, low-density lipoprotein; SBP, systolic blood pressure; ULSAM, Uppsala Longitudinal Study of Adult Men.

(Figure 4, online supplemental table 4)In the observational part of the study, BMI and SBP were related to incident atrial fibrillation in the univariate analyses. In the multivariate model, BMI and SBP, but also diabetes and LDL, were significantly related to atrial fibrillation, the latter two in a negative fashion. Of those four, SBP and BMI were the risk factor being most closely related to atrial fibrillation with equally low p values.

In the MR part of the study, SBP, BMI and smoking were related to atrial fibrillation. In the multivariate MR model, only BMI and SBP were significantly related to atrial fibrillation. Of those four, BMI was the risk factor being most closely related to atrial fibrillation, followed by SBP.

## Discussion

The novelty of this study is that we investigated all the traditional cardiovascular risk factors for the four major CVDs in a systematic and comprehensive fashion both univariate and multivariate, and both in a long-term observational setting, as well as using MR.

SBP was related to all four CVDs in a powerful, multivariate fashion both in the observational and MR part of the study, thus being the overall most important risk factor for a major CVD. While the important role of SBP is well-known from epidemiological studies since decades, data from MR studies for all major CVDs have been lacking.

BMI was the second most powerful risk factor from an overall perspective, since BMI was related to all the four CVDs, except ischaemic stroke, in the multivariate models.

Several studies have used the MR technique to investigate the causal role of obesity, measured by BMI, on CVDs. In early studies, a polygenetic gene score with a limited number of SNPs for BMI was related to CHD,^9^ and heart failure.^10^ In a later study using multiple SNPs as genetic instruments, the early findings were reproduced and, in addition, a causal relationship with ischaemic stroke was demonstrated.^11^ Following these early findings, the causal role of obesity for CHD, heart failure and stroke have been reproduced by other studies, and also a causal role of obesity regarding atrial fibrillation has been demonstrated.^26–28^ We did also find BMI to be significantly related to ischaemic stroke in the univariate models used both in the observational and MR part, but BMI was not significant in the multivariate models. Since multivariate MR was not used in the above cited MR studies, this fact might explain the discrepant findings.

Smoking was an important risk factor for ischaemic stroke and heart failure, both in observational and MR analyses in the multivariate models. Two previous studies have shown smoking to be causally related to ischaemic stroke,^29 30^ but MR studies regarding smoking and heart failure are lacking.

LDL-cholesterol was of major importance for myocardial infarction, but was not significant for other CVDs in the multivariate models. HDL-cholesterol and triglycerides were mainly significant in the univariate models, but not in the multivariate fashion. Although it has uniformly been shown that LDL-cholesterol is causally related to CHD and ischaemic stroke.^12–14 31^ The role of HDL-cholesterol as causal risk factor has previously been disproved.^12 13^ A causal action of elevated triglycerides has been proposed.^12 14^ In addition, genetic instruments for LDL-cholesterol and triglycerides have been associated with heart failure incidence,^14^ but the causal role of lipids for atrial fibrillation is little known. Since multivariate MR was not generally used in these other MR studies, this fact might explain the discrepant findings regarding the role of LDL-cholesterol versus stroke and heart failure. LDL-cholesterol was in fact related to stroke and heart failure in the univariate MR models.

Diabetes was mainly a causal risk factor for incident myocardial infarction and heart failure in our multivariate models. Several studies have shown that diabetes, high glucose levels or elevated haemoglobin A1c are causally related to CHD and ischaemic stroke,^32–35^ but we could not find any previous MR studies on diabetes as a risk factor for heart failure or atrial fibrillation. Again, since multivariate MR was not generally used in these other MR studies, this fact might explain the discrepant findings regarding the role of diabetes versus stroke. In the univariate MR models, diabetes was related to ischaemic stroke.

The observational part of the study did not disclose any unexpected findings. We did not find LDL-cholesterol and smoking to be associated with ischaemic stroke, as reported by others.^3^ At the baseline investigation in the early 70s, half of the cohort were active smokers. The smoking rate then rapidly declined during the coming two decades to 20%.^36^ Therefore, information on smoking only at baseline in the 1970s might not be sufficient information to capture the full burden of smoking during four decades. Also, LDL-cholesterol declined by >1 mmol/L during the subsequent two decades due to general changes in dietary preferences in Sweden during the 70s and eighties. Other risk factors did not change so dramatically during the follow-up period.

### Strengths and limitations

Strengths of the observational part of the study include a homogeneous cohort, prospective collection of risk factors, zero lost to follow-up and a long follow-up period, allowing evaluation of effects of risk factors over a prolonged period of time, a situation that resembles the MR situation. Another strength is that we could evaluate associations of all risk factors for all four CVDs in both the observational and MR setting in a comprehensive way, and that we used both univariate and multivariate models in both the observational and MR part. The major limitation of our observational study is that we only studied Swedish men, rendering an unknown generalisability to women and other ethnic groups.

While the evaluation of risk factors in the observational part of the study was performed in a single sample, the MR part used multiple GWASs for the different risk factors and four GWASs for the outcomes. The GWASs for risk factors have different strengths as instrumental variables, just as the GWASs for the outcomes show different power in finding genetic loci for the outcomes. Therefore, the MR part of the study is more heterogeneous and the causal estimates are not readily comparable between the risk factors or the outcomes.

## Conclusion

By combining long-term observational data with genetic data, we show that the impact and causal role of specific established cardiovascular risk factors varies between different major CVDs. SBP was causally related to all four cardiovascular outcomes, and was therefore the overall most important risk factor.

## Data Availability

Data are available on reasonable request. Due to Swedish law and the Ethical Committee permission, health data at the individual level cannot be made available online as an open source for the public. Data are however available by a request sent to the corresponding author.

## Data availability statement

Due to Swedish law and the Ethical Committee permission, health data at the individual level cannot be made available online as an open source for the public. Data are however available by a request sent to the corresponding author.
