# Supplementary material for: Impact of risk factors for major cardiovascular diseases: a comparison of life-time observational and Mendelian randomisation findings
Source: Open Heart. 2021 Sep 13;8(2):e001735. doi: 10.1136/openhrt-2021-001735 (PMC8438838; doi:10.1136/openhrt-2021-001735)
Supplement: Supplementary data [file openhrt-2021-001735supp001.pdf]

BMJ Publishing Group Limited (BMJ) disclaims all liability and responsibility arising from any reliance placed on this supplemental material which has been supplied by the author(s)

SUPPLEMENTAL MATERIALS

THE IMPACT OF RISK FACTORS FOR MAJOR CARDIOVASCULAR DISEASES - A COMPARISON OF LIFE-TIME OBSERVATIONAL AND MENDELIAN RANDOMIZATION FINDINGS

Lars Lind, Martin Ingelsson, Johan Sundström, Johan Ärnlöv

Contents

Supplementary Table 1.......2
Supplementary Table 2.......4
Supplementary Table 3.......6
Supplementary Table 4.......8

Lind L, *et al. Open Heart* 2022; 8:e001735. doi: 10.1136/openhrt-2021-001735

Supplementary Table 1. Sensitivity Mendelian randomization analyses of associations of risk factors with coronary heart disease. MR Egger estimates are given both for a univariate (risk factors analyzed one by one) and a multivariate model (including all risk factors in the same model). Number of independent SNPs used as instruments is given in parenthesis for each risk factor.

	Test	Estimate	95%CI lower	95%CI higher	*P*-value
	**Smoking** (n=97)				
Univariate	MR Egger	2.98	0.93	9.51	0.064
Univariate	Weighted median	2.32	1.62	3.34	<0.0001
Univariate	Heterogeneity	Q=107			0.15
Multivariate	MR Egger	1.55	1.15	2.09	0.0043
	**Diabetes** (n=40)				
Univariate	MR Egger	1.39	1.07	1.80	0.013
Univariate	Weighted median	1.13	1.06	1.21	0.00031
Univariate	Heterogeneity	Q=89			0.0001
Multivariate	MR Egger	1.07	1.02	1.13	0.010
	**BMI** (N=79)				
Univariate	MR Egger	1.56	1.22	1.99	0.00034
Univariate	Weighted median	1.49	1.29	1.72	<0.0001
Univariate	Heterogeneity	Q=103			0.018
Multivariate	MR Egger	1.18	1.06	1.32	0.0021
	**HDL** (n=84)				
Univariate	MR Egger	1.01	.085	1.20	0.91
Univariate	Weighted median	0.92	0.83	1.03	0.16
Univariate	Heterogeneity	Q=279			<0.0001
Multivariate	MR Egger	0.93	0.84	1.04	0.21
	**Triglycerides** (n=51)				
Univariate	MR Egger	1.10	0.94	1.29	0.24
Univariate	Weighted median	1.21	1.08	1.35	0.00069
Univariate	Heterogeneity	Q=144			<0.0001
Multivariate	MR Egger	1.03	0.90	1.18	0.64

Lind L, *et al. Open Heart* 2022; 8:e001735. doi: 10.1136/openhrt-2021-001735

	**LDL** (n=76)				
Univariate	MR Egger	1.54	1.36	1.75	<0.0001
Univariate	Weighted median	1.52	1.40	1.66	<0.0001
Univariate	Heterogeneity	Q=245			<0.0001
Multivariate	MR Egger	1.36	1.25	1.48	<0.0001
	**Systolic blood pressure** (n=228)				
Univariate	MR Egger	1.03	1.01	1.06	0.0049
Univariate	Weighted median	1.03	1.03	1.04	<0.0001
Univariate	Heterogeneity	Q=488			<0.0001
Multivariate	MR Egger	1.03	1.02	1.04	<0.0001

Supplementary Table 2. Sensitivity Mendelian randomization analyses of associations of risk factors with ischemic stroke. MR Egger estimates are given both for a univariate (risk factors analyzed one by one) and a multivariate model (including all risk factors in the same model). Number of independent SNPs used as instruments is given in parenthesis for each risk factor.

	Test	Estimate	95%CI lower	95%CI higher	*P*-value
	**Smoking (n=97)**				
Univariate	MR Egger	1.33	0.48	3.69	0.57
Univariate	Weighted median	1.52	1.11	2.07	0.0084
Univariate	Heterogeneity	Q=150			0.0635
Multivariate	MR Egger	1.50	1.12	2.01	0.0059
	**Diabetes** (n=42)				
Univariate	MR Egger	1.17	0.94	1.47	0.16
Univariate	Weighted median	1.17	1.10	1.25	<0.0001
Univariate	Heterogeneity	Q=70			0.0022
Multivariate	MR Egger	1.07	1.02	1.13	0.011
	**BMI** (n=78)				
Univariate	MR Egger	0.96	0.72	1.28	0.79
Univariate	Weighted median	1.07	0.92	1.25	0.35
Univariate	Heterogeneity	Q=130			0.0001
Multivariate	MR Egger	0.99	0.89	1.10	0.80
	**HDL** (n=87)				
Univariate	MR Egger	1.13	1.01	1.27	0.047
Univariate	Weighted median	1.01	0.93	1.10	0.81
Univariate	Heterogeneity	Q=122			0.0057
Multivariate	MR Egger	0.97	0.87	1.07	0.51
	**Triglycerides** (n=53)				
Univariate	MR Egger	0.95	0.84	1.07	0.37
Univariate	Weighted median	1.01	0.91	1.10	0.91
Univariate	Heterogeneity	Q=61			0.14
Multivariate	MR Egger	1.02	0.90	1.16	0.73

	**LDL** (n=77)				
Univariate	MR Egger	1.17	1.06	1.29	0.0017
Univariate	Weighted median	1.12	1.03	1.21	0.0047
Univariate	Heterogeneity	Q=145			<0.0001
Multivariate	MR Egger	1.02	0.94	1.11	0.60
	**Systolic blood pressure** (n=229)				
Univariate	MR Egger	1.03	1.01	1.05	0.019
Univariate	Weighted median	1.03	1.02	1.03	<0.0001
Univariate	Heterogeneity	Q=342			<0.0001
Multivariate	MR Egger	1.02	1.02	1.03	<0.0001

Supplementary Table 3. Sensitivity Mendelian randomization analyses of associations of risk factors with heart failure. MR Egger estimates are given both for a univariate (risk factors analyzed one by one) and a multivariate model (including all risk factors in the same model). Number of independent SNPs used as instruments is given in parenthesis for each risk factor.

	Test	Estimate	95%CI lower	95%CI higher	*P*-value
	**Smoking** (n=123)				
Univariate	MR Egger	1.48	0.63	3.50	0.36
Univariate	Weighted median	1.86	1.43	2.41	<0.0001
Univariate	Heterogeneity	Q=164			0.0051
Multivariate	MR Egger	1.28	1.05	1.57	0.016
	**Diabetes** (n=42)				
Univariate	MR Egger	1.07	0.91	1.26	0.40
Univariate	Weighted median	1.07	1.02	1.12	0.0051
Univariate	Heterogeneity	Q=73			0.0010
Multivariate	MR Egger	1.05	1.02	1.08	0.0011
	**BMI** (n=78)				
Univariate	MR Egger	1.57	1.21	2.03	0.00057
Univariate	Weighted median	1.67	1.46	1.92	<0.0001
Univariate	Heterogeneity	Q=167			<0.0001
Multivariate	MR Egger	1.57	1.46	1.68	<0.0001
	**HDL** (n=86)				
Univariate	MR Egger	0.94	0.84	1.06	0.33
Univariate	Weighted median	0.94	0.87	1.02	0.14
Univariate	Heterogeneity	Q=189			<0.0001
Multivariate	MR Egger	0.98	0.92	1.06	0.67
	**Triglycerides** (n=53)				
Univariate	MR Egger	1.17	1.05	1.31	0.0055
Univariate	Weighted median	1.16	1.07	1.25	0.00038
Univariate	Heterogeneity	Q=91			0.0004
Multivariate	MR Egger	1.02	0.93	1.12	0.65

Lind L, *et al. Open Heart* 2022; 8:e001735. doi: 10.1136/openhrt-2021-001735

	**LDL** (n=77)				
Univariate	MR Egger	1.11	1.02	1.21	0.012
Univariate	Weighted median	1.10	1.01	1.2	0.023
Univariate	Heterogeneity	Q=198			<0.0001
Multivariate	MR Egger	1.02	0.96	1.08	0.54
	**Systolic blood pressure** (n=231)				
Univariate	MR Egger	1.02	0.99	1.03	0.068
Univariate	Weighted median	1.02	1.01	1.03	<0.0001
Univariate	Heterogeneity	Q=361			<0.0001
Multivariate	MR Egger	1.02	1.01	1.02	<0.0001

Supplementary Table 4. Sensitivity Mendelian randomization analyses of associations of risk factors with atrial fibrillation. MR Egger estimates are given both for a univariate (risk factors analyzed one by one) and a multivariate model (including all risk factors in the same model). Number of independent SNPs used as instruments is given in parenthesis for each risk factor.

	Test	Estimate	95%CI lower	95%CI higher	*P*-value
	**Smoking** (n=125)				
Univariate	MR Egger	1.61	0.70	3.69	0.26
Univariate	Weighted median	1.01	0.79	1.29	0.91
Univariate	Heterogeneity	Q=190			0.0001
Multivariate	MR Egger	1.14	0.88	1.47	0.33
	**Diabetes** (n=42)				
Univariate	MR Egger	0.99	0.86	1.13	0.85
Univariate	Weighted median	0.98	0.94	1.03	0.43
Univariate	Heterogeneity	Q=59			0.024
Multivariate	MR Egger	0.96	0.92	1.01	0.12
	**BMI** (n=77)				
Univariate	MR Egger	1.16	0.96	1.39	0.11
Univariate	Weighted median	1.35	1.21	1.52	<0.0001
Univariate	Heterogeneity	Q=100			0.029
Multivariate	MR Egger	1.23	1.12	1.35	<0.0001
	**HDL** (n=86)				
Univariate	MR Egger	1.01	0.92	1.12	0.79
Univariate	Weighted median	0.99	0.93	1.06	0.83
Univariate	Heterogeneity	Q=168			0.0001
Multivariate	MR Egger	0.92	0.84	1.01	0.077
	**Triglycerides** (n=51)				
Univariate	MR Egger	1.04	0.91	1.18	0.55
Univariate	Weighted median	0.98	0.91	1.06	0.61
Univariate	Heterogeneity	Q=157			<0.0001
Multivariate	MR Egger	0.93	0.83	1.04	0.20

Lind L, *et al. Open Heart* 2022; 8:e001735. doi: 10.1136/openhrt-2021-001735

	**LDL** (n=79)				
Univariate	MR Egger	1.03	0.96	1.10	0.38
Univariate	Weighted median	0.97	0.92	1.03	0.33
Univariate	Heterogeneity	Q=141			<0.0001
Multivariate	MR Egger	1.01	0.94	1.08	0.83
	**Systolic blood pressure** (n=231)				
Univariate	MR Egger	1.01	0.98	1.02	0.93
Univariate	Weighted median	1.01	1.01	1.02	<0.0001
Univariate	Heterogeneity	Q=442			<0.0001
Multivariate	MR Egger	1.01	1.00	1.01	0.0055

Lind L, *et al. Open Heart* 2022; 8:e001735. doi: 10.1136/openhrt-2021-001735
